# Socio-epidemiological and therapeutic profile of patients infected by HIV in 2011 at Tunis (Tunisia)

**DOI:** 10.1186/1471-2334-14-S2-P46

**Published:** 2014-05-23

**Authors:** A Mrabet, MT Khoufi, A Yaakoubi, MR Kamoun, A Ben Hamida

**Affiliations:** 1 Faculty of Medicine of Tunis, Tunis, Tunisia

## Introduction

Tunisia began, since 2004, with UNAIDS collaboration, strategic planning against HIV infection and STD to figure out the epidemic dynamic and his determinants, reduce the risk of transmission of STD/HIV among the vulnerable persons, and reduce the mortality and morbidity in people living with HIV(PLHIV) and families by improvement of whole support. Our aim was to trace the socio-epidemiological profile and the treatment of persons infected by HIV in 2011 in Tunis.

## Methods

A retrospective study of 56 patients infected by HIV whose treatment began in 2011 in the reference medical department of HIV in Tunis. We included all patients whose HIV infection was confirmed by Western Blot and whose medical support began in the disease stage during the first medical consultation according to the clinical and biological criteria of CDC.

## Results

Population mean age was 38.4 years. PLHIV faced unemployment problems. Half of patients are destitute, 10.8% are students and 62% live lonesome. Imprisonment was found in 3.6% of patients. 79% of PLHIV have Tunisian nationality and benefit of free tritherapy and medical support. 50% made at least one travel abroad. The foreign PLHIV are from sub-saharan African countries. Risks behaviors are multiple, as non protected heterosexual intercourse (68%). The discovery of HIV infection occurs in stage A (62%), C3 (21%). All eligible patients to a highly active antiretroviral therapy (HAART) received the treatment. 12% have co-infection HIV – Hepatitis B and/or C. VHC was found in injection drug users (p<10-3). Opportunistic infections were observed in 30%. Good observance was notified in 70%, in the first year of treatment. The poor observance was due to unavailability of HAART and to the denial of a binding treatment. Four deaths were notified: males, singles, C3 stage in beginning of treatment.

## Conclusion

PLHIV are a part of the population that cumulates vulnerability factors which increases the risks for reaching further stages of the AIDS and for poor medical support.

